# Synergistic Effect and Mechanism of Nano-C-S-H Seed and Calcium Sulfoaluminate Cement on the Early Mechanical Properties of Portland Cement

**DOI:** 10.3390/ma16041575

**Published:** 2023-02-13

**Authors:** Ruifeng Tang, Dawei Sun, Zhaojia Wang, Ziming Wang, Suping Cui, Wenxu Ma, Mingzhang Lan

**Affiliations:** 1 Faculty of Materials Manufacture, Beijing University of Technology, Beijing 100124, China; 2 State Key Laboratory of Soild Waste Utilization and Energy-Saving Building Materials, Beijing Building Materials Research Institute, Beijing 100041, China

**Keywords:** nano-C-S-H seed, calcium sulfoaluminate cement, early strength, synergistic effect, ettringite

## Abstract

The combined utilization of mineral accelerators and nano-seeding materials is a novel method to promote the early strength of cement-based materials. In this paper, the effects of nano-C-S-H seed (NCS) on the early compressive strength of the Portland cement (PC)– calcium sulfoaluminate cement (CSA) binder were investigated. The results showed that NCS and CSA synergistically contributed to the early strength of PC. In detail, a 326.3% increase in the 10 h compressive strength of PC paste was obtained through the addition of NCS (2 wt%) and CSA (5%) in common. This was higher than the sum of the increases observed with the single additions of CSA (157.9%) or NCS (87.6%), with the same above dosage, in PC. Meanwhile, the early strength enhancement effects of NCS and CSA, when used together in PC, lasted longer than the effects of either used alone. Moreover, the synergetic effect mechanism was analyzed by isothermal calorimeter, QXRD, TGA, MIP, and SEM techniques. The calorimetry, XRD, and TGA results demonstrated that the synergistic mechanism was associated with the synergistic promotion effects of CSA and NCS on the hydrates. The fast hydration of CSA produced large amounts of ettringite and also consumed partial free water to promote the performance of the seeding effect of NCS which, simultaneously, further accelerated the precipitation of C-S-H gel and CH. The high alkie environment was also beneficial for the continuous generation of ettringite. In addition, the results of MIP and SEM measurements showed that the micro-filling effect of NCS significantly optimized the pore structure of a PC-CSA blend-hardened paste. Thus, the synergistic strength enhancement effects of CSA and NCS on PC were attributed to the matching of the promotion of hydration generation and the optimization of pore structures in hardening cement paste. The results of this article provide a new approach to achieving the rapid development of the early strength of cementitious materials, with potential applications in precast concrete and low-temperature construction.

## 1. Introduction

In many projects, such as those involving precast concrete and winter construction, the rapid early strength development of cement is often required [1,2]. For example, many factories choose the steam curing method to accelerate the early strength of cement, aiming to improve the demolding speed of precast elements. However, steam curing not only causes heat damage to the interior of the concrete, reducing the long-term strength and durability, but also increases the energy consumption and emission of carbon dioxygen [3,4,5]. The use of chemical accelerators is another common method used to improve the early strength of cementitious materials. However, the early strength promotional effects of most traditional inorganic/organic accelerators based on calcium salts or alcohol amine are limited. Additionally, they can react with cement clinker to form other substances, leading to a decrease in the long-term strength [6,7,8,9,10]. As the most widely-used cement-based material, Portland cement (PC) is likely to see a continued use in building and civil engineering construction, on a large scale, for the foreseeable future. Thus, the healthy and rapid development of the early strength of PC, under the premise of a low energy consumption, has become an issue of great concern in current academic and engineering fields. Due to this, the combined utilization of mineral accelerators and accelerating admixtures in PC for the purpose of improving its early strength was an inevitable trend.

The use of mineral accelerators, such as CA, C_12_A_7_, and C_4_A_3_$\overset{—}{S}$, for PC is considered a promising method to promote the healthy development of early strength [11,12,13,14,15,16]. Calcium sulfoaluminate cement (CSA) is a type of high early strength cementitious material with ye’elimate (C_4_A_3_$\overset{—}{S}$) and belite (C_2_S) as the main minerals. It mainly contains the following three early reactions (Equations (1)–(3)) at different conditions [17,18,19,20,21,22,23]. Among these, CaSO_4_, Ca(OH)_2_, and Al(OH)_3_ are marked as C$\overset{—}{S}$, CH, and AH_3_, respectively. Thus, the rapid production of ettringite (C_3_A·3C$\overset{—}{S}$·32H, AFt) is the main reason for the high early strength of CSA. As the other primarily mineral phase of CSA, β-C_2_S exhibits a slow dissolution and hydration rate and offers basically no contribution to the early strength of CSA. However, some recent works in the literature showed that the addition of impurity ions in the preparation of CSA caused the lattice distortion of β-C_2_S, which improved the dissolution rate and hydration activity of belite. According to previous works in the literature, the addition of B^3+^, K^+^, Na^+^, P^5+^, and Fe^3+^ caused the lattice distortion of β-C_2_S, transforming it into α-C_2_S (which was beneficial to the acceleration of the dissolution rate) and improving the 28-day hydration degree of belite as well as the strength properties of cement [24,25,26]. Recently, Izadifar et al. developed an upscaling approach, the atomistic kinetic Monte Carlo (KMC), to achieve comprehension and quantify the mesoscopic forward dissolution rate of C_2_S. They found that a layer-by-layer dissolution mechanism was responsible for the slow reactivity of belite clinker. Additionally, the introduction of crystal defects, namely, cutting the edges at two crystal boundaries, increased the overall average dissolution rate by a factor of 519 [27]. This could be beneficial to applications of high-belite cement, facilitating low energy consumption and reducing carbon dioxide emissions. The hydration products of belite are calcium silicate hydrate (C-S-H, xCaO-ySiO_2_-zH_2_O, C-S-H) gel and portlandite (CH). Among the hydrates of CSA, portlandite is the most soluble hydration phase. The dissolution rates of portlandite differ for different surfaces, comprehensively, as it is highly formed after hydration. Izadifar et al. found that the initial dissolution process of portlandite took place from the edges, sides, and facets of 010 or 010 of the crystal morphology [28]. However, both the crystal form of belite and the dissolution rate of portlandite had little influence on the early hydration of CSA. Therefore, this paper did not focus on these issues.

(1) $$C_{4}A_{3}\overset{—}{S}+2\;C\overset{—}{S}+38\;H_{2}O\;\to \;C_{3}A\cdot 3C\overset{—}{S}\cdot 32H+2AH_{3}\;(\mathrm{in}\;\mathrm{the}\;\mathrm{presence}\;\mathrm{of}\;\mathrm{gypsum})$$

(2) $$C_{4}A_{3}\overset{—}{S}+8\;C\overset{—}{S}+6\;\mathrm{CH}+90\;H_{2}O\;\to \;3C_{3}A\cdot 3C\overset{—}{S}\cdot 32H\;(\mathrm{in}\;\mathrm{the}\;\mathrm{presence}\;\mathrm{of}\;\mathrm{gypsum}\;\mathrm{and}\;\mathrm{CH})$$

(3) $$C_{4}A_{3}\overset{—}{S}+18H_{2}O\to C_{3}A\cdot C\overset{—}{S}\cdot 12H+2AH_{3}\;(\mathrm{in}\;\mathrm{the}\;\mathrm{absence}\;\mathrm{of}\;\mathrm{gypsum})$$

Compared to PC, manufacturing CSA cement emits 50–85% less CO_2_ due to the lower limestone requirement and lower calcination temperature (1300 °C) [17]. Thus, the partial replacement of PC by CSA not only leads to high early strength but also reduces the environmental impact of the emission of CO_2_ from cement production. Many papers have confirmed that a blend of PC and CSA could combine the advantages of both, e.g., excellent early strength, great dimensional stability, good durability, low shrinkage, and green features [29,30,31,32,33,34,35,36,37,38], effectively creating a potential cementitious material for precast concrete and winter construction. Moreover, in the blend of PC and CSA, the increase in CSA percentage facilitates the rapid early strength development of this blend [33]. However, the high-quality bauxite (the raw material of ye’elimite) gradually reduces after the development of more than 50 years, leading to the cost of CSA elevating [29]. In addition, the excessively rapid generation of ettringite might aggravate the pore structure, damaging the hardening density of the PC-CSA matrix [34]. Therefore, the percentage of CSA is often limited to less than 10% of the total cementitious material, which keeps the costs competitive and compactness of PC-CSA binder but may limit the degree of early strength enhancement.

As is known to all, calcium silicate hydrate (xCaO-ySiO_2_-zH_2_O, C-S-H) gel is the main hydrate (approximately 60% by mass) of PC, which is responsible for the strength properties of hardened cement [39]. The nano-C-S-H particles are synthetic nanoparticles with the same chemical composition as C-S-H gel, compared to other nano-particles, such as nano-SiO_2_ and nano-CaCO_3_. Thus, the synthetic nano-C-S-H is the most ideal nucleation matrix for C-S-H gel [40]. In the early hydration process, they can provide more additional crystallization sites for hydration products, thus reducing the activation energy of crystallization and accelerating their growth. Thus, the nano-C-S-H particles were called nano-C-S-H seeds (NCS) due to the similar process to seeding [41]. The common method synthesis of NCS includes the pozzolanic method, sol-gel method, and chemical precipitation method [42]. Among them, nano-C-S-H prepared by chemical precipitation in the presence polymers can effectively solve the agglomeration problem of nanoparticles and maximize the performance of the seeding effect of NCS. It has become the main preparation method of NCS at present [43].

The synthetic NCS addressed the above shortcoming in the traditional accelerator via the seeding effect [44,45,46,47,48]. Bost et al. [40] compared the effect of NCS with other accelerating additions (e.g., CaCl_2_, Ca(NO_3_)_2_, CaCO_3_, and so on) on the hydration heat of cement. They found that the NCS showed the highest hydration heat flows. Meanwhile, the long-term strength of cement is not sacrificed by the addition of NCS due to the same chemical composition as the C-S-H gel. Scientific research also found that the accelerating effect of NCS compensated for the low hydration activity of supplementary cementitious materials. For example, Kanchananson et al. [49] analyzed the effect of NCS on the early strength development of slag blend cement. The results showed that the NCS not only promoted the early crystallization of C-S-H but also stimulated the pozzolanic reaction of slag, resulting in obtaining a higher early strength. The same effects on the fly ash blend cement were also published in the research of Plank et al. [50]. This indicated that a synergistic hydration promotion existed between NCS and different cement minerals. However, it was difficult to find a clear description of the effect of the combined utilization of CSA and NCS on the early strength of PC in the published literature. Therefore, although the combined use of the CSA mineral accelerator and NCS chemical accelerator was a new idea for the early strength enhancement of PC, more studies were needed to demonstrate the synergistic effect of both.

As a result, in this paper, a PC-CSA binder consisting of PC clinker (90%), CSA clinker (5%), and anhydrite (5%) was designed, due to the cost competition and the greatest potential for applications of this ration of the PC-CSA binder [34]. Meanwhile, different dosages of NCS on the early compressive strength of the PC-CSA binder were investigated and compared to its effect in PC, aiming to explore the synergistic effect of CSA and NCS. Moreover, the synergistic working mechanism of NCS and CSA was studied via isothermal calorimetry, XRD, TGA, MIP, and SEM measurements. The results of this study are expected to contribute to a carbon reduction in the cement industry by providing assistance for projects with high requirements for early strength, such as precast concrete and winter construction.

## 2. Materials and Methods

### 2.1. Materials

**Figure 1:**
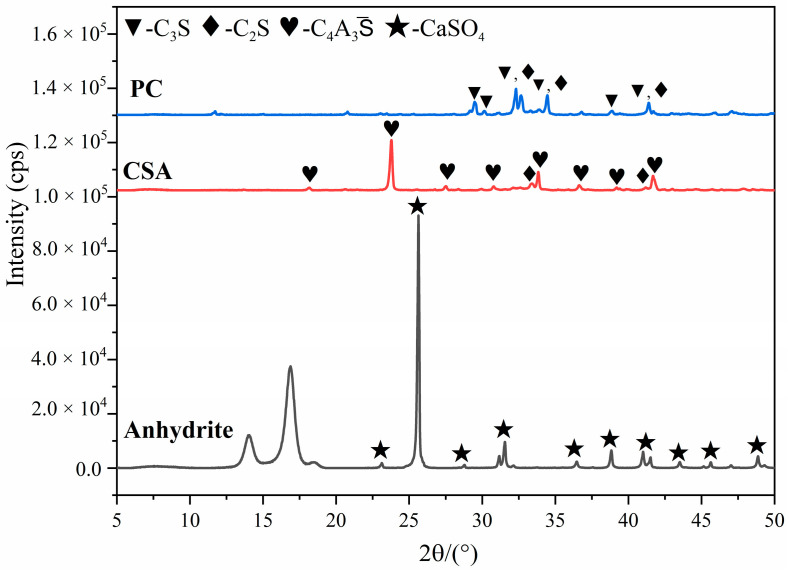
XRD patterns of raw materials.

The Portland cement (PC) clinker used in this paper was obtained from Beijing Liulihe Cement Plant (Beijing, China), and the calcium sulfoaluminate cement (CSA) clinker and anhydrite were obtained from China Building Materials Academy Co. Ltd. (Beijing, China). Their chemical compositions and mineral compositions were measured using XRF and XRD, which were presented in Table 1 and Table 2 and Figure 1. According to Table 2, it can be found that there is a large difference in the mineral composition between the PC clinker and the CSA clinker. Obviously, the basicity of the CSA clinker is lower than that of the PC clinker because the hydration rate of C_2_S is far slower than that of C_3_S. Thus, the addition of CSA in PC to composite PC-CSA not only obtained the high early strength but also reduced the basicity of the cement, which was beneficial to the improvement of the ability of sulfate resistance [51,52]. The Blaine method was used to measure the surface area of the PC clinker and CSA clinker with the result of 355 m^2^/g and 370 m^2^/g. In the synthesis of NCS, the calcium and silicon sources were Ca(NO_3_)_2_·4H_2_O and Na_2_SiO_3_·5H_2_O, respectively, both of which were purchased from Beijing Chemical Co. Ltd. (Beijing, China). The commercial PCE was purchased from China Building materials Academy Co. Ltd. (Beijing, China). NaOH and HNO_3_ used for pH adjustments were purchased from Sinopharm Chemical Reagent Co., Ltd. (Shanghai, China).

The NCS suspension was prepared by the co-precipitation method by adding Ca(NO_3_)_2_ and Na_2_SiO_3_ solutions to the PCE solution according to Land G [48]. The initial mole ratio of calcium to silicon was 1.7. Firstly, 20 g of the PCE stabilizers were diluted to 5.6% with deionized water and subsequently adjusted the pH value to 11.5 ± 0.5 with aqueous 30% NaOH. Then, the PCE dilution was poured into a 1000 mL round-bottom flask with N_2_ atmosphere at 25 °C. Subsequently, 45.7 g of Ca(NO_3_)_2_ solution with a concentration of 25% and 49.5 g of Na_2_SiO_3_ solution with a concentration of 10% were added dropwise to the PCE solution and kept stirring at 600 rpm. The drop was finished in 2 h. Meanwhile, the pH value of the reaction solution was kept at 11.5 ± 0.5 by adding HNO_3_ or NaOH solutions according to the feedback from the pH electrode in the round-bottom flask. The final milky NCS suspension has a solids content of 10 wt%. The NCS suspensions were used as in the TEM (JEM 2100, Jeol Co., Shizuoka, Japan) and DLS (ZS 900, Malvern instruments Ltd., Marvin City, UK) measurements. Additionally, some of the NCS suspensions were washed with deionized water and dried at 40 °C for 3 d to convert to powder. These powders were used for XRD (D8 Advance, Shimadzu Co., Kyoto, Japan) and FT-IR (Vertex 70 Spectrometer, Brooke Co., Werther, German) measurements.

**Figure 2:**
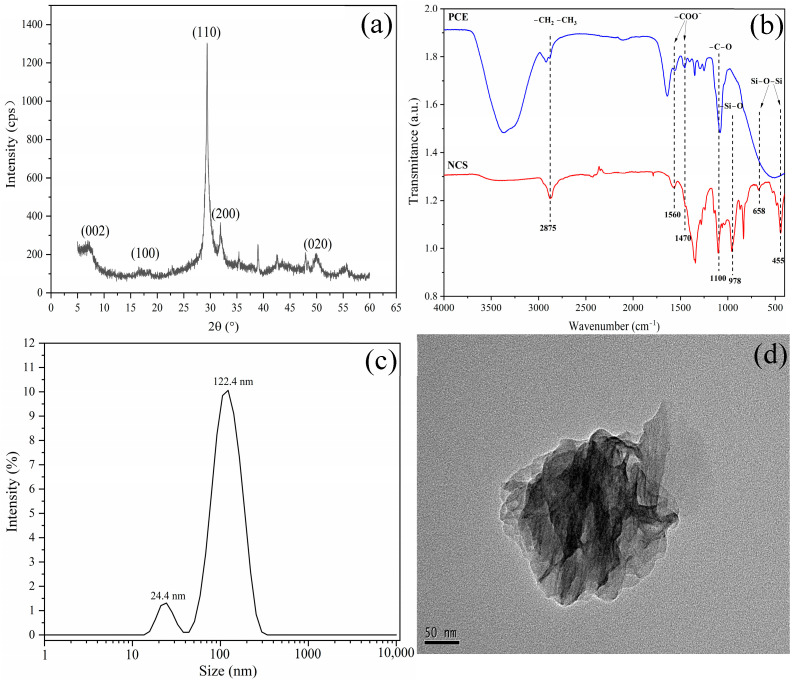
Characterization of NCS: (**a**) XRD pattern, (**b**) FT-IR spectra: blue line represents PCE, red line represents NCS, (**c**) particles size distribution of NCS, (**d**) morphology of NCS.

The XRD pattern (Figure 2a) showed that the diffraction peaks of synthesized CNS with (hk0) indices (002), (110), (200), and (020) were found at 2θ = 7.0°, 29.0°, 32°, 49.7°, respectively, which was in good accordance with tobermorite [53]. The peak at 17° distributed diffusely and lowly, demonstrating that carbonization existed in the process of NCS synthesis [46]. The FT-IR spectra of NCS and PCE are presented in Figure 2b. For NCS, the wide hydroxyl group peaks observed at 3500 and 1640 cm^−1^ were related to the crystal water in the structure of C-S-H. The peaks at 455 cm^−1^ and 658 cm^−1^ were associated with the stretching vibration of Si-O-Si, and the peak at 978 cm^−1^ wavenumbers corresponded to the Si-O stretching vibration of tobermorite. In addition, the symmetric and asymmetric stretching vibration peaks of COO- appeared at 1470 cm^−1^ and 1560 cm^−1^ wavenumbers, and the peak at the 2875 cm^−1^ wavenumber corresponded with the stretching vibration peaks of −CH_2_ and −CH_3_ which were also observed in the FT-IR curve of NCS, which was consistent with the characteristic spectra of PCE. The particle size distribution and morphology of NCS was shown in Figure 2c,d. The DLS test results confirmed the nanometer properties of the synthesized NCS. As shown in Figure 2c, the particle size distribution diagram revealed a bimodal size distribution, containing two fractions, ~24 nm and ~122 nm. Among them, NCS with a size of about 122 nm accounted for the majority. It can be seen from Figure 2d that a single NCS particles was a nanoparticle with a layered and tinfoil shape, with an average size of about 150 nm, which is consistent with the result of the DLS test and the morphology previously recorded in the literature [54,55]. The above results confirmed the nano-scale NCS synthesis successfully.

### 2.2. Cement Paste Specimens

The mixed proportions of different binders are given in Table 3. The sample of PC and PC-CSA represented the PC binder and PC-CSA binder, respectively. The dosage of NCS suspensions (as solid) was 0.5%, 1.0%, and 2.0% by the mass of the blend of PC clinker, CSA clinker, and anhydrite phase. The water-to-binder ratio (w/c) was 0.3. The water in the NCS suspension was part of the mixed water. Therefore, in order to ensure that the w/c ratio of each group of samples was 0.3, the content of water added to the cement paste samples with different dosages of NCS suspension was different. A supplementary explanation is contained is this paper. The detailed mix proportions were shown in Table 4. The specified time-cured paste samples were crushed and placed in the isopropanol solution for 24 h to terminate hydration. The filtered samples were placed in a vacuum drying oven at 60 °C for 3 d. One part was fragmented into small pieces to be tested for compressive strength, MIP, and SEM analysis; the other was ground into powders to be tested for XRD and TGA analysis.

### 2.3. Methods

#### 2.3.1. Compressive Strength

The cement pastes were shaped in the steed molds of 20 mm × 20 mm × 20 mm and cured in the curing chamber with a constant temperature (20 ± 1 °C) and 95% humidity. In this experiment, 10 h, 1 d, 3 d, and 28 d specimens were submitted to compressive strength measurements. A group of 6 test blocks had the average value as the compressive strength. The detailed mix proportions are shown in Table 4.

#### 2.3.2. Hydration Heat

A TAM AIR-08 isothermal calorimeter was employed to measure the hydration kinetics of different binders at 25 °C. The proportion of the pastes is shown in Table 5. The detailed operation was as follows: 3 g cement powders were first added into the glass ampoule. Then, the injector containing water and NCS suspensions was connected to the ampoule. After that, the ampoule and injector connector were fed into the isothermal calorimeter. After the baseline went flat, the aqueous solution was quickly injected into the ampoule and stirred for 3 min to commence the measurement.

#### 2.3.3. XRD Analysis

Using XRD analysis and the Rietveld refinement technique, the phase composition of cement paste was quantitatively investigated. XRD patterns were employed a D8 Advance diffractometer at 40 kV and 100 mA with CuKa radiation. The scanning range of 5~70° with a step length of 0.01°. Corundum (α-Al_2_O_3_, 10%) was used as an internal standard, and mixed with the stop cement powder. The Rietveld calculation was carried out by using TOPAS Academic V5.

#### 2.3.4. TGA Analysis

The thermogravimetric instrument (STA449F3, Selbu, Bavaria, Germany) was employed to measure the composition and relative content of hydration products of different binders. The hydrated sample powder weighed about 20 mg and was placed in a corundum crucible. The sample together with the Al_2_O_3_ crucible was elevated from room temperature of 25 °C to 800 °C at N_2_ with a controlled heating rate of 10 °C/min. The content of chemically bound water (*BW*) and portlandite (*CH*) was calculated according to Equation (4) and Equation (5) [56], respectively.

(4) $$W_{BW}=\frac{W_{50}-W_{550}}{W_{550}}\times 100$$

(5) $$W_{CH}=\frac{\left(W_{400}-W_{500}\right)}{W_{550}}\times \frac{74}{18}\times 100\;$$

where the *W*_50_, *W*_400_, *W*_500_, and *W*_550_ was the weight of specimens at 50 °C, 400 °C, 500 °C, and 550 °C.

#### 2.3.5. MIP Analysis

The Auto Pore V 9600 mercury piezometer (Micromeritics, Norcross, GA, USA) was used to investigate the pore structure of cement paste at early age hydration. The mercury piezometer has a minimum pressure of 0.0014 MPa and a maximum pressure of 420 MPa. Pore size can be measured anywhere from 0.003 to 400 μm.

#### 2.3.6. SEM Analysis

The microstructure of different hardening binders was observed by the field emission scanning electron microscope (SU 8020, Hitachi, Co., Tokyo, Japan).

## 3. Results and Discussion

### 3.1. Compressive Strength

**Figure 3:**
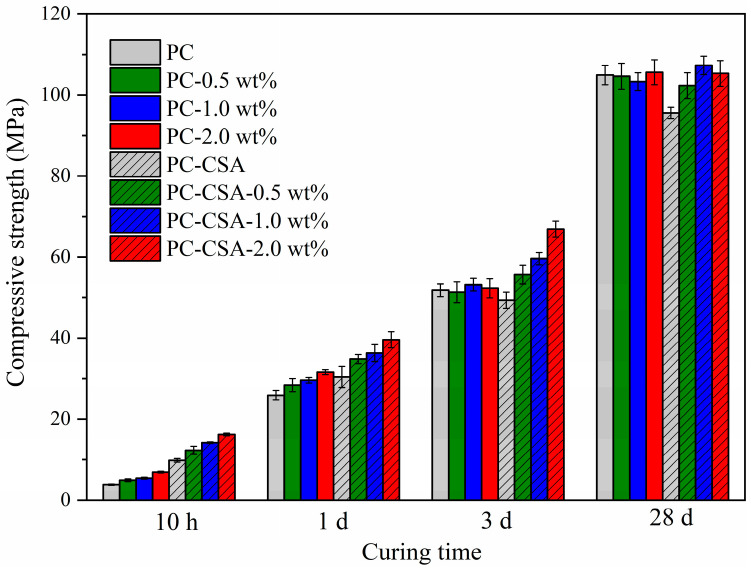
Compressive strength of different binders.

The influence of NCS on the early compressive strength of PC and PC-CSA paste was investigated first, and the detailed data are shown in Figure 3 and Table 6. At 10 h, the compressive strength of PC paste was gradually improved with the increase in the NCS dosage. While the dosage of NCS was 0.5 wt%, 1.0 wt%, and 2.0 wt%, the compressive strength of pastes at 10 h of curing were 4.9 MPa, 5.4 MPa, and 6.9 MPa, which increased by 28.9%, 42.1%, and 81.6% compared to the PC sample, respectively (Table 6). This was related to the nucleation effect of NCS, which reduces the barriers of C-S-H gel formation in the early hydration and accelerates the formation of C-S-H gel [46,47]. Thus, the 10 h compressive strength of PC was increased obviously. However, the difference in the compressive strength of the samples with and without NCS gradually decreased after the 1 d curing age. This phenomenon showed that the strength enhancement of NCS on PC was limited and mainly within the 1 d curing age. However, NCS had no adverse effect on the growth of cement strength in the late stages. For example, at 28 d, the compressive strength of PC, PC-0.5 wt%, PC-1.0 wt%, and PC-2.0 wt% were 104.9 MPa, 104.6 MPa, 103.3 MPa, and 105.6 MPa, respectively. This was in agreement with the previous literature [47].

As shown in Figure 3, the 10 h compressive strength of PC-CSA was significantly higher than that of PC, which arrived at 9.8 MPa, due to the fast generation of AFt [30]. However, with the prolonged curing time, the growth rate of compressive strength of PC-CSA gradually slowed down. At 3 d and 28 d, there was even a slight decrease in the compressive strength of PC-CSA compared to PC. The possible reason was that the rapid generation of AFt might cause an uneven distribution of pores in the PC-CSA matrix, which in turn reduced the denseness of cement-hardened paste [34]. This was not conducive to the development of the strength of cementitious materials.

We can see clearly from Figure 3 that the 10 h compressive strength of PC-CSA was gradually increased by the increased dosage of NCS, which performed a similar trend as in PC. However, the compressive strength of PC-CSA containing NCS was significantly higher than that of PC containing the same dose of NCS. This may be related to the synergistic effect between NCS and CSA. For instance, in comparison to the PC, the compressive strength of PC-CSA-2.0 wt% increases by 326.3% at 10 h, which was higher than the sum of the promotion effect of PC-2.0 wt% (81.6%) and PC-CSA (157.9%) (Table 6). Meanwhile, the strength loss of PC-CSA at 3 d and 28 d was compensated by the addition of NCS. In detail, at the age of 3 d, the compressive strengths of PC-CSA-0.5 wt%, PC-CSA-1.0 wt%, and PC-CSA-2.0 wt% were 55.7 MPa, 59.6 MPa, and 66.9 MPa, respectively, which were 7.5%, 15.1%, and 29.2% higher than PC. At this curing time, the addition of NCS (0.5 wt%, 1.0 wt%, 2.0 wt%) or CSA (5%) alone in PC almost did not contribute to the growth of the compressive strength. At 28 d, the compressive of PC-CSA-0.5 wt%, PC-CSA-1.0 wt%, and PC-CSA-2.0 wt% was 102.3 MPa, 107.3 MPa, and 105.3 MPa, respectively. Although there was no obvious improvement compared with the PC, it compensated for the slow compressive strength growth of PC-CSA (97.6 MPa) at 28 d. It demonstrated that the addition of NCS to PC-CSA not only performed a higher early strength enhancement, but also obtained the more stable middle–late-stage strength growth rates.

**Figure 4:**
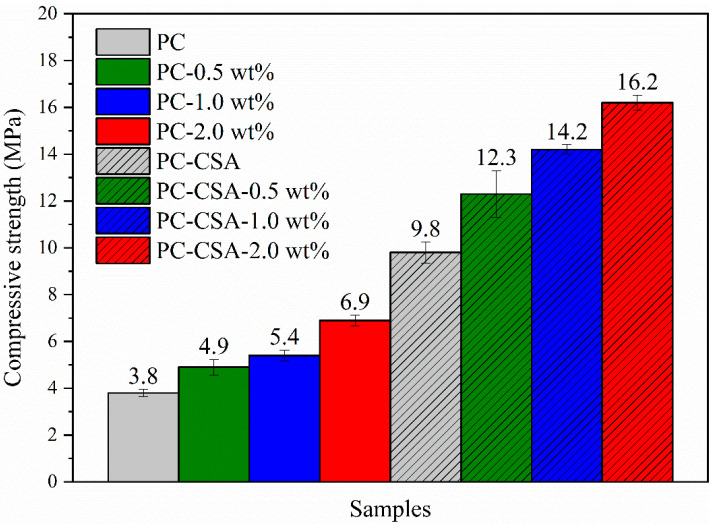
The 10 h compressive strength of different samples.

**Figure 5:**
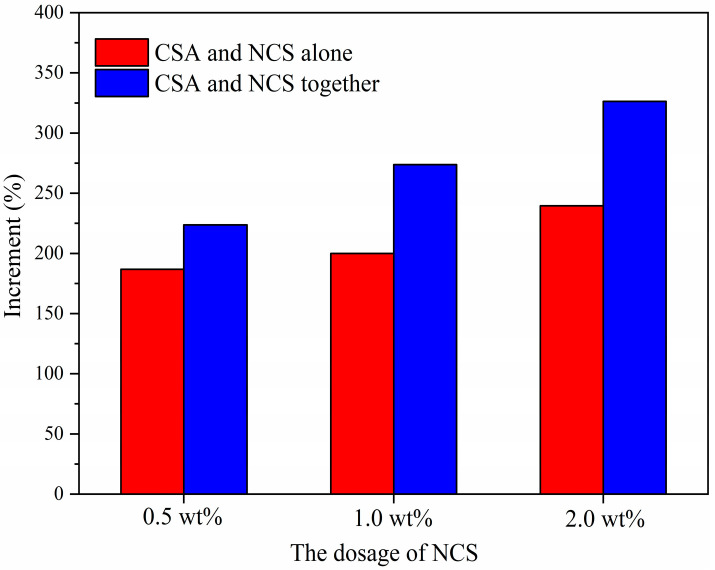
The 10 h compressive strength growth ratio of PC with the addition of CSA or/and NCS.

In order to determine whether there was a synergistic effect of CSA and NCS on the early strength enhancement of PC, we compared the 10 h strength increase sum brought by the addition of CSA and NCS alone with the 10 h strength increase brought by the addition of both, according to the data in Table 6 and Figure 4. Surprisingly, the gap between separate and co-addition was observed clearly in Figure 5 that the 10 h compressive strength increment of the addition of NCS and CSA in common was higher than the strength increase sum of the addition of NCS and CSA alone, regardless of the dosage of NCS. It demonstrated that the synergistic strength enhancement of CSA and NCS took place in PC; the detailed data are shown in Table 6. Obviously, the high addition percentage (2.0 wt%) of NCS performed a stronger synergistic effect with CSA. Therefore, to better explain the mechanism of NCS and CSA synergistically enhancing the early strength of PC, the high addition percentage (2.0 wt%) of NCS was used in order to augment the effects of the hydration mechanisms involved and to better detect minor changes in the samples during the hydration. Thus, four groups of samples, PC, PC-2.0 wt%, PC-CSA, and PC-CSA-2.0 wt%, were selected for subsequent testing.

### 3.2. Hydration Kinetics

**Figure 6:**
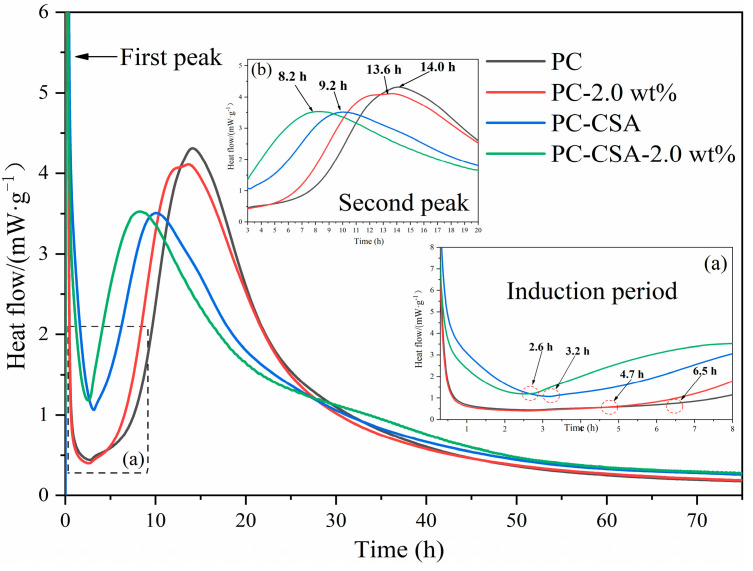
Hydration heat flow of different binders. (**a**) Second peaks, (**b**) induction period.

**Figure 7:**
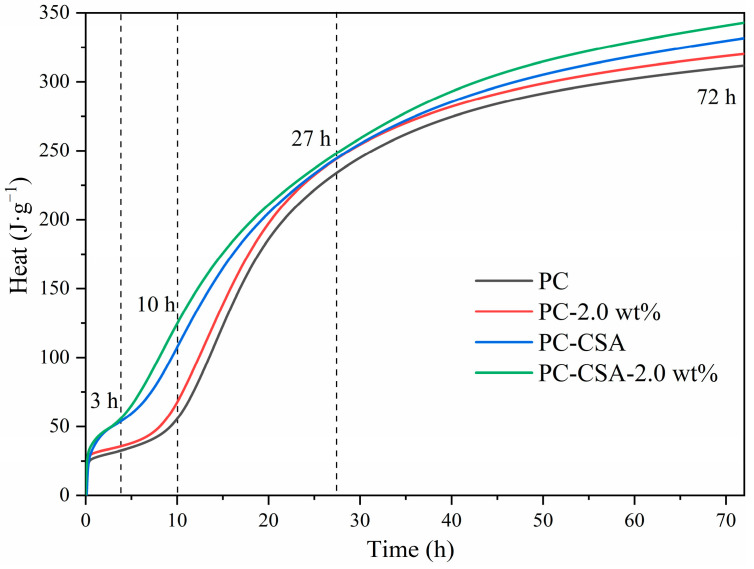
Cumulative hydration heat of different binders.

The hydration heat evolution of different binders was investigated via isothermal calorimetry to reveal the synergistic effect mechanism of CSA and NCS in PC; the detailed data are shown in Figure 6 and Figure 7. In the neat PC, the first peak (from left to right) appeared around 6 min after contact of the cement particles with water due to the wetting of cement grains and the partial formation of ettringite [57,58]. The induction period occurred between approximately 0.6 and 6.5 h. After 6.5 h, the accelerated period appeared due to the accelerated formation of C-S-H gel and the second peak appeared at around 14 h. What followed was the deceleration period. As can be seen from Figure 6, the heat flow curves changed at different stages with the addition of NCS or/and CSA.

With the addition of 2.0 wt% NCS in PC, the shorter induction period and the earlier second heat flow peak observed in Figure 6a,b compared to pure PC. This was mainly related to the seed nucleation effect of NCS. According to the dissolution–precipitation theory, the gap in the induction period was mainly controlled by the nucleation sites [59,60,61]. The well dispersed NCSs act as the extra nucleation sites for the C-S-H gel, which diminished the cement clinker dissolution barrier and crystalline energy barrier of hydration products, accelerating the hydration of PC, which was in agreement with previous reports [46,47].

The heat flow curve of PC-CSA was significantly different from that of neat PC, which was due to the different compositions of the cement minerals involved in early hydration between PC and CSA [14]. Compared to PC, the PC-CSA has a shorter induction period and a more advanced second heat flow peak due to the fast generation of ettringite. Another difference between PC-CSA and PC was that the PC-CSA had a lower second heat flow peak than the PC samples. The possible reasons are as follows. Scrivener et al. [57] showed that the second heat flow peak kinetics were controlled with the grow space of C-S-H “needles”, which was generated from the surface of cement particles and extended outwards during the hydration process. When the hydrates mesh dense with each other, there was no growth space for needles on the surface of the grain, the inner C-S-H gel started to form, and the heat flow curves up to the peak value [62]. Thus, the fast generation of ettringite in PC-CSA would limit the growth space of the C-S-H “needle”, promoting the inner C-S-H to start to form. In comparison to the C-S-H “needle”, the inner C-S-H grows slowly. Thus, in the literature, it had sometimes been reported that the hydration of C_3_S was retarded by the generation of ettringite in the blend of PC and CSA [63].

With the addition of NCS and CSA in PC based on the above dosage, it was marked to PC-CSA-2.0 wt%. The hydration heat evolution of PC-CSA-2.0 wt% was similar to the PC-CSA. However, it had a shorter induction period and earlier second heat flow peak than PC-CSA (Figure 6), which performed the fastest hydration heat flow among these four binders in the early period. According to the literature [34], the fast generation of ettringite could consume more free water to accelerate the precipitation of C-S-H gel in the blend of PC and CSA. It suggested that the seeding effect of NCS and the fast generation of ettringite might promote the dissolution of C_3_S together and increase the SiO_3_^2−^ concentration, resulting in the rapid precipitation of C-S-H gel and CH, launching the acceleration period of PC-CSA-2.0 wt% synergistically. During the subsequent heat flow deceleration period, a “shoulder” was observed in the heat flow curve of PC-CSA-2.0 wt%. It might also correlate to the synergistic effect of CSA and NCS. On the one hand, the generation of inner C-S-H gel was promoted by the dispersed NCS in the pore solution, leading to the increased generation of CH. On the other hand, the high content of CH was also beneficial to the continued formation of ettringite according to the reaction (2). Therefore, a higher hydration heat flow between 27 and 50 h was observed in PC-CSA-2.0 wt%, which was in agreement with the compressive strength test.

More details were available from the cumulative hydration heat curves of different binders (Figure 7). Before 3 h, PC-CSA and PC-CSA-2.0 wt% had similar curves and cumulated heat faster than PC and PC-2.0 wt%. It demonstrated that the early strength enhancement of both PC-CSA and PC-CSA-2.0 wt% within 3 h was obtained from the rapid formation of ettringite. Between approximately 3 and 10 h, PC-CSA-2.0 wt% started to release more heat of hydration, which was related to the synergistic promotion of hydration by NCS and CSA. However, the gap of cumulative hydration heat between PC-CSA and PC-2.0 wt% tended to decrease during the hydration time of 10 h and 27 h, which was related to the retard effect of ettringite on the C-S-H growth [63]. For the PC-CSA-2.0 wt%, the synergistic effect of CSA and NCS in the early hydration period reduced this retardation effect, maintaining a slightly higher cumulative hydration heat. After 27 h, the second fast increase in the cumulative hydration heat of PC-CSA and PC-CSA-2.0 wt% was observed. It suggested that the retardation of ettringite disappeared, and the C_3_S mineral restarted to generate C-S-H gel at the same speed as PC. The cumulative heat of hydration increased more rapidly for PC-CSA-2.0 wt% compared to PC-CSA, suggesting that there was also a synergistic promotion of ettringite and C-S-H gel by CSA and NCS during the deceleration period of the hydration process, which confirmed the heat flow analysis. In general, the trend of hydration heat of different binders was similar to the compressive strength, which suggested that the synergistic early strength enhancement of CSA and NCS was related to the generation rate of different hydrates. The variations in the hydration and microstructure of different binders would be the subject of the following analysis.

### 3.3. XRD Analysis

**Figure 8:**
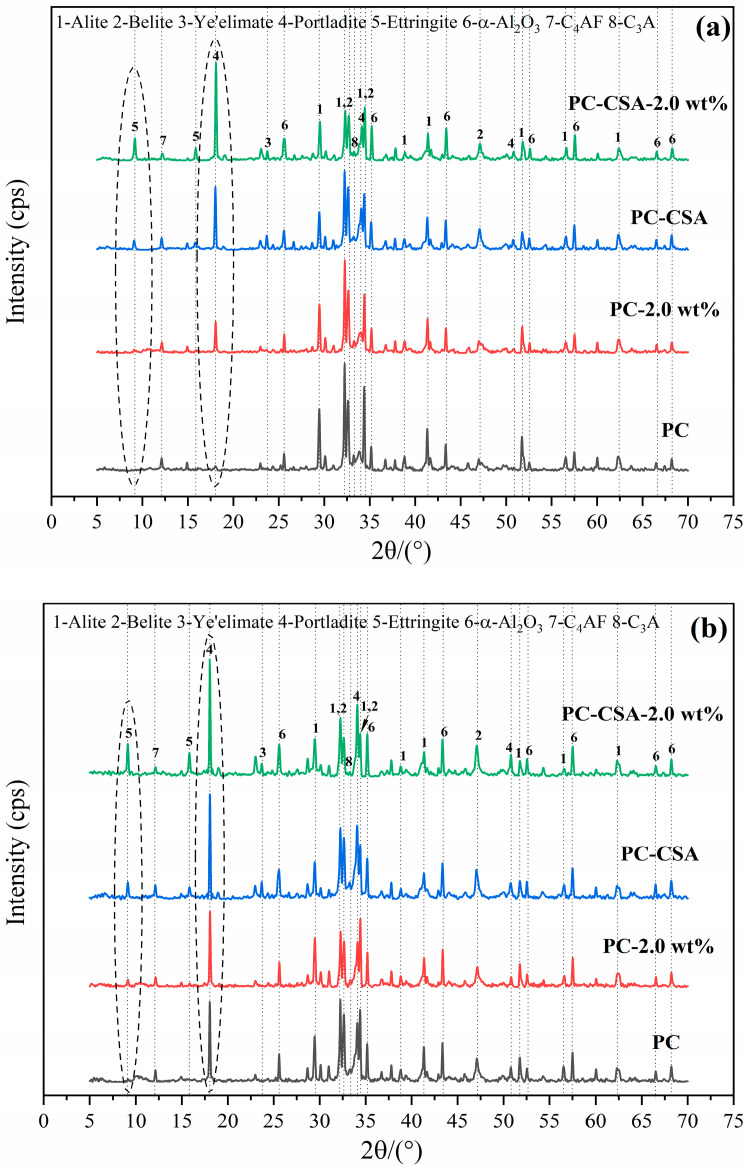

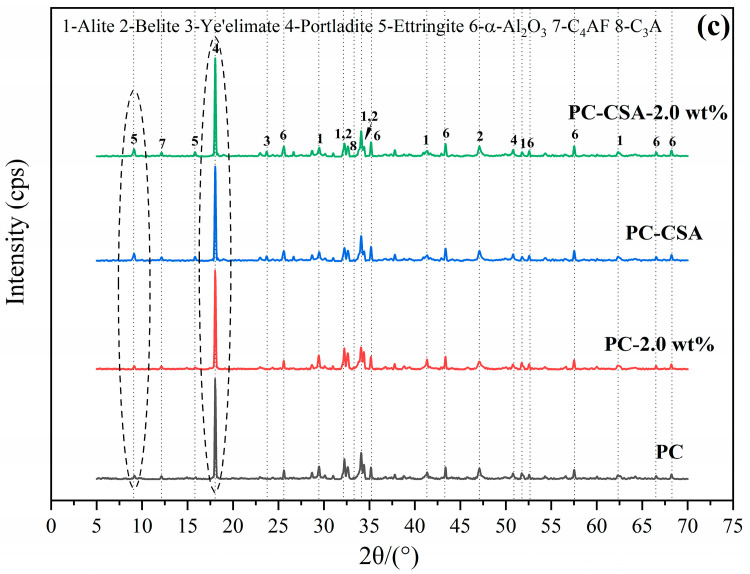
XRD patterns of different binders at different curing times: (**a**) 10 h; (**b**) 1 d; (**c**) 3 d.

**Figure 9:**
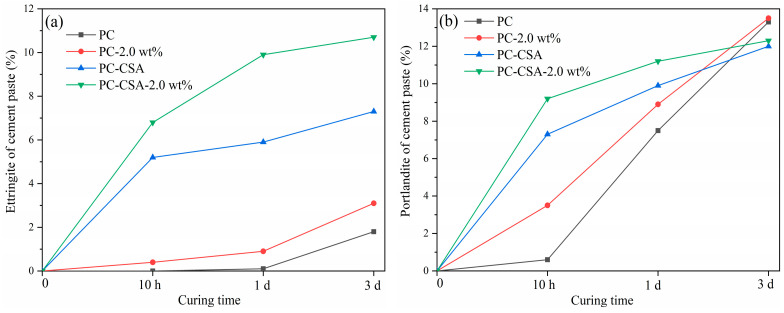
The content of hydrates in different binders at different curing times: (**a**) ettringite; (**b**) portlandite.

Complementary to the hydration heat analysis, the quantitative XRD technique was used to investigate the development of early hydrates of different binders. The data are shown in Figure 8 and Figure 9. Corundum was used as an internal standard sample. The obtained XRD data show that the early reaction cement mineral was C_3_S and C_3_A in PC, while it was C_3_S, C_4_A_3_$\overset{—}{S}$, and C_3_A in PC-CSA (Figure 8). In general, the crystal hydrates which formed were similar in PC with the addition of NCS or CSA, which was the ettringite and portlandite (CH). However, the content of both existed varies in different binders (Figure 9). The main peak of ettringite (2θ = 9°) and the diffraction peak of CH (2θ = 18°) of different binders was selected for comparison.

In the PC-2.0 wt% sample, an intense reflection characteristic for the CH phase was observed at 10 h, which corroborated the seeding effect of NCS. In PC-CSA, the stronger intensity diffraction peak of ettringite was observed due to the hydration of CSA. Among the four binders, the most intense diffraction peak of ettringite and CH at 10 h (Figure 8a) was observed in the PC-CSA-2.0 wt%, indicating that CSA and NCS could synergistically promote the formation of hydrates by their respective characteristics during the early hydration stage. At 1 d and 3 d, although the PC-CSA-2.0 wt% maintained the high-intensity diffraction peak of ettringite, there was a tendency for the diffraction peak intensity gap to converge for CH with different binders (Figure 8b,c). Combined with the data in Figure 9, it suggested that with the hydration of PC-CSA-2.0 wt%, the CH would be consumed to maintain the continuous generation of ettringite. Thus, the content of ettringite of PC-CSA-2.0 wt% was higher within 3 d all the time, while CH increased and then decreased. The XRD techniques confirmed the analysis of hydration heat.

### 3.4. TGA Analysis

**Figure 10:**
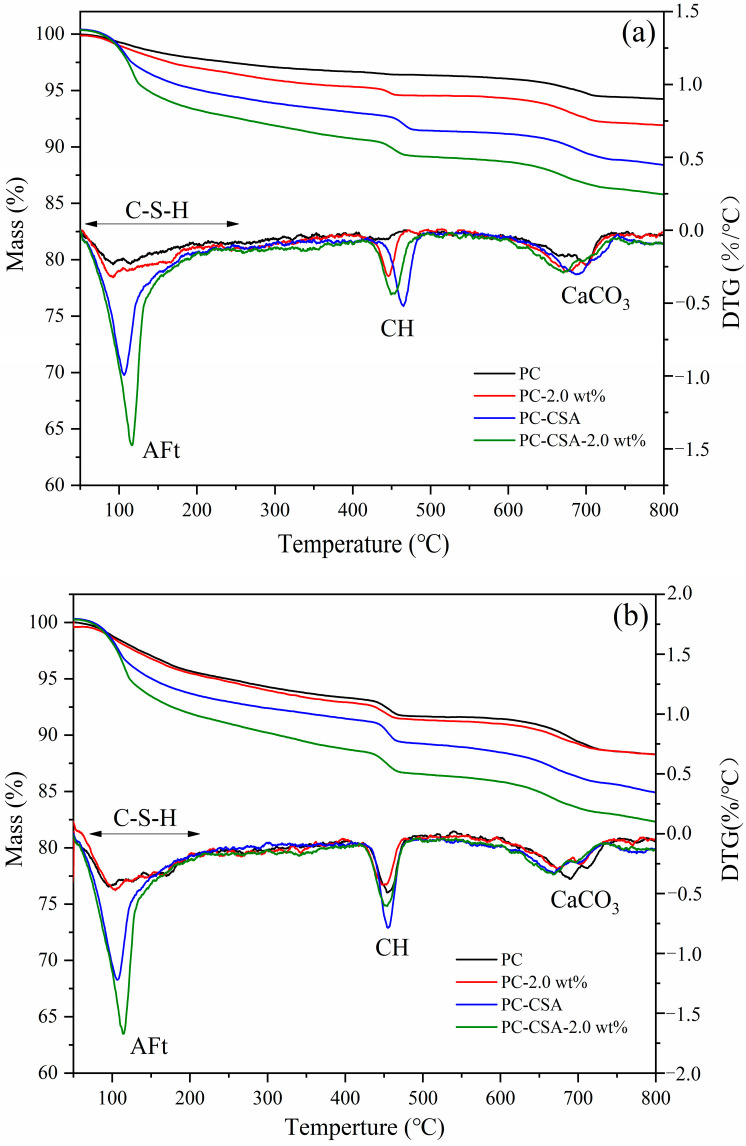

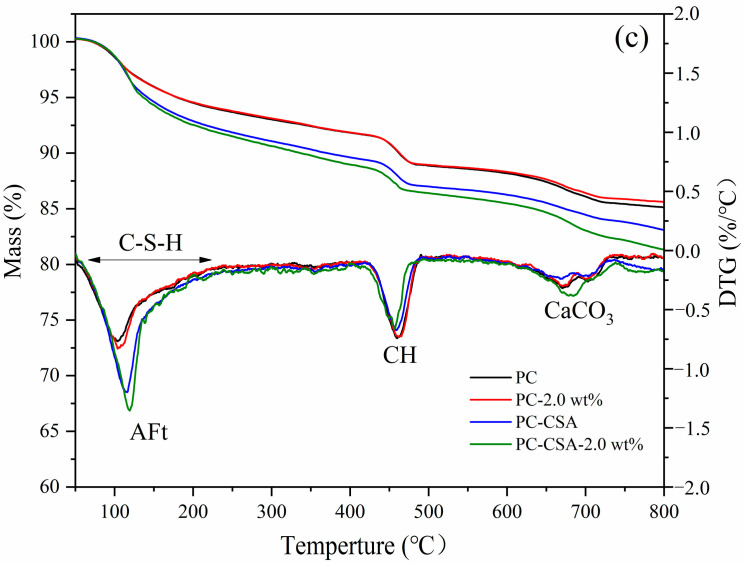
Thermogravimetric analysis of cement (**a**) 10 h; (**b**) 1 d; (**c**) 3 d.

TGA analyses were used to identify the amorphous and crystalized hydrates formed in the different binders, as well as their content, to confirm the results by QXRD. The DTG signals were obtained from the TG measurements, which are shown in Figure 10. There were mainly three peaks in the DTG curves. The weight-loss peak at around 50–200 °C was related to the decomposition of free water, C-S-H gel, ettringite, and hemicarbonate [33]. Therefore, it was hard to accurately calculate the content of ettringite relying on the TGA analyses. The weight-loss peak at 400–500 °C was associated with the formation of CH. The weight-loss peak at 600–800 °C might be related to the decomposition of the carbonates. The content of bound water (BW) and portlandite (CH) calculated from the mass loss curve are listed in Table 7, which was mainly used to identify the hydration degree of different binders.

According to the TG curve in Figure 10a, the increasing order of a mass loss of cement paste was PC, PC-2.0 wt%, PC-CSA, and PC-CSA-2.0 wt% at 10 h. The increased content of BW and CH in PC-CSA-2.0 wt% compared to the other three samples can be observed in Table 7. It confirmed that CSA and NCS could synergistically promote hydrate production during early hydration, which was responsible for the early strength enhancement of PC. At 3 d, although the content of BW in PC-CSA-2.0 wt% was still more than that of PC-2.0 wt% and PC-CSA, the content of CH is lower. It is possible that the increased generation of ettringite in PC-CSA-2.0 wt% caused the increased consumption of CH in the hydration process when hydration was prolonged. It was consistent with the hydration heat and XRD analysis. The detailed information on the contents of BW and CH is listed in Table 7.

### 3.5. Pore Structure Analysis

**Figure 11:**
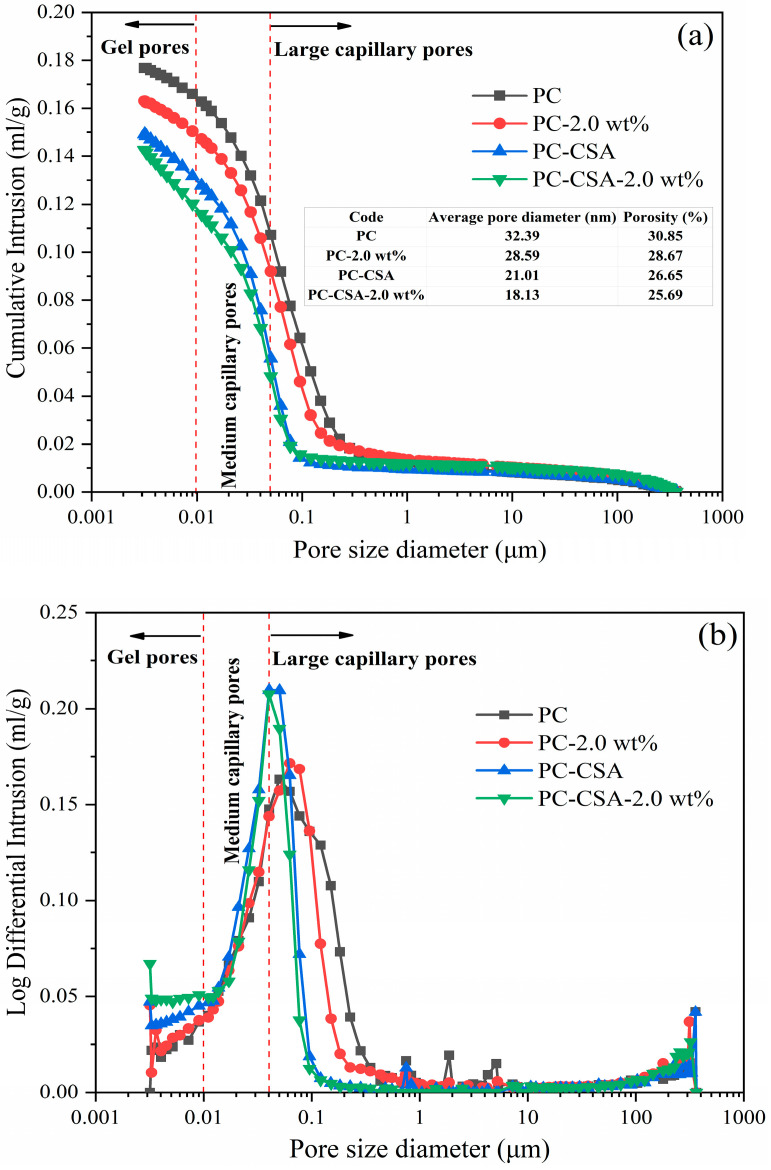
The pore structure of cement at 10 h curing: (**a**) cumulative intrusion; (**b**) differential intrusion.

**Figure 12:**
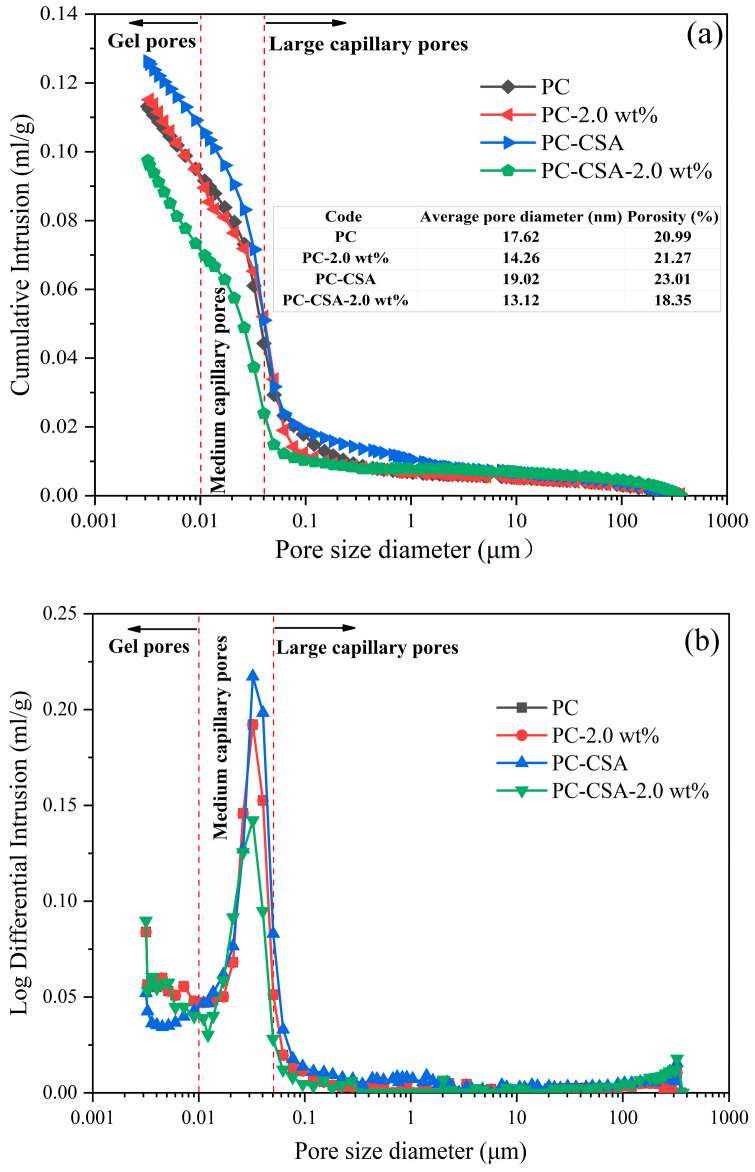
The pore structure of cement at 3 d curing: (**a**) cumulative intrusion; (**b**) differential intrusion.

Many papers have shown that the voids increase around CSA grains due to the fast generation of ettringite, damaging the strength development of the PC-CSA binder [38,63,64]. As a consequence, the MIP measurement was employed to explore the pore structure of different binders at 10 h and 3 d, with the findings being provided in Figure 11 and Figure 12. As we can see from Figure 11a, both the pore volume intruded by mercury and the critical radius decreased with the addition of NCS and CSA in PC at 10 h. Clearly, the addition of CSA and NCS in common had more benefits in the decrease in the pore volume intruded of cement because the smallest pore volume was observed at PC-CSA-2.0 wt%. Meanwhile, at 10 h, the distribution of the pore entry size in Figure 11b showed a sharp peak in PC-CSA and PC-CSA-2.0 wt% for both, which became broader in PC and PC-2.0 wt%. In addition, in comparison with the other three samples, the number of large capillary pores (0.05–10 μm) of PC-CSA-2.0 wt% was reduced, while the number of medium capillary pores (0.01–0.05 μm) and gel pores (0–0.01 μm) was increased [65]. According to the above data, it can be concluded that the CSA had a strong contribution to reducing the porosity of cement paste at an early age. Because the fast ettringite which formed constructed the dense crystal skeleton and promoted the hardening of cement paste. Thus, the porosity of both PC-CSA and PC-CSA-2.0 wt% was lower than the PC and PC-2.0 wt% for both. Specifically, in the PC-CSA-2.0 wt% sample, the NCS has a synergistic effect with CSA to accelerate the C-S-H gel form, in turn filling the space around the crystal skeleton and reducing the porosity of the paste. Therefore, in comparison with the other three samples, PC-CSA-2.0 wt% had the lowest porosity at 10 h, which was in agreement with the compressive strength measurement.

Figure 12 showed the pore structure of different binders at 3 d. The order of porosity at 3 d was different from at 10 h. PC-CSA had a more cumulative pore volume than the other three blends due to the rapid production of ettringite, resulting in the uneven distribution of the hydrates [63]. As a result, the compressive strength of the PC-CSA sample was lower than the PC sample at 3 days. The cumulative pore volume of both PC and PC-2.0 wt% were similar at 3 d, which was in agreement with the compressive strength. Meanwhile, the lowest cumulative pore volume was observed in the PC-CSA-2.0 wt% sample, suggesting that excess NCS might fill the pore in the hardened cement paste of the PC-CSA binders [66]. In the differentiated curve in Figure 12b, a similar pore size distribution for the four blended cement pastes was observed, which concentrated on the medium capillary pores. However, the peak of PC-CSA-2.0 wt% was narrower and shorter than the other three samples. It indicated that PC-CSA-2.0 wt% had the denser cement matrix at 3 d. It suggested that the NCS not only act as the seeds to promote the nucleation and growth of C-S-H gel but also performed the micro-filling effect in hardening the cement paste, reducing the porosity. Thus, the compressive strength of PC-CSA-2.0 wt% was higher than PC-2.0 wt% and PC-CSA at 3 d.

### 3.6. SEM Analysis

**Figure 13:**
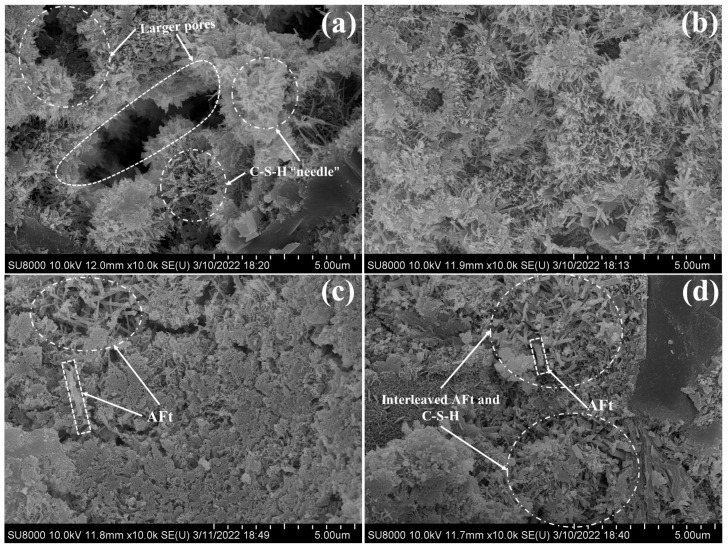
Morphology of different binders after curing 10 h, (**a**) PC; (**b**) PC-2.0 wt%; (**c**) PC-CSA; (**d**) PC-CSA-2.0 wt%.

**Figure 14:**
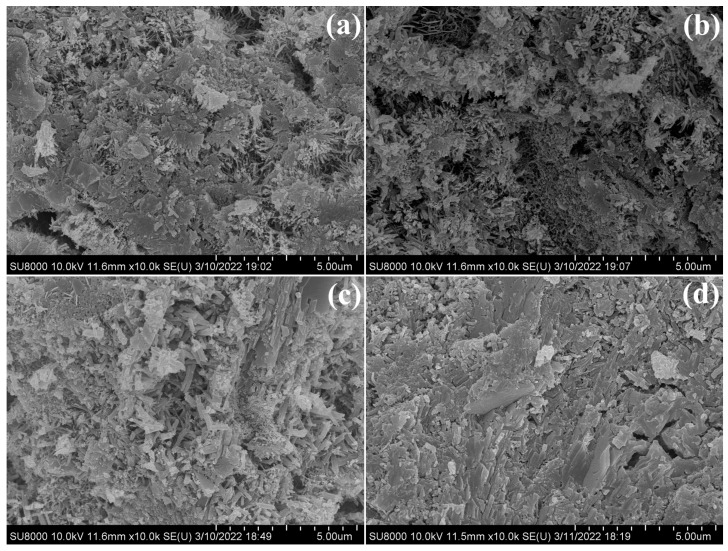
Morphology of different binders after curing 3 d, (**a**) PC; (**b**) PC-2.0 wt%; (**c**) PC-CSA; (**d**) PC-CSA-2.0 wt%.

The samples PC, PC-2.0 wt%, PC-CSA, and PC-CSA-2.0 wt% were analyzed by SEM to determine the effect of CSA and NCS on the evolution of the hydrates microstructure at early hydration. Similar hydrates morphologies were observed in PC and PC-2.0 wt% at 10 h (Figure 13a,b). The morphology was needle-like C-S-H. However, the morphology of PC was looser than PC-2.0 wt%, corresponding to the larger pores. As shown in Figure 13b, more needle-like C-S-H was formed in the PC-2.0 wt% and filled the pores. It was related to the seeding effect of NCS. In PC-CSA and PC-CSA-2.0 wt%, the cement matrix seems to be denser due to the generation of rod-like ettringite at 10 h (Figure 13c,d). Compared to PC-CSA, the distribution of ettringite in PC-CSA-2.0 wt% was more uniform and interleave with C-S-H. Moreover, more C-S-H and CH can be observed in Figure 13d. It revealed the synergistic early strength enhancement effect of CSA and NCS to generate more hydrates and dense the morphology of PC-CSA-2.0 wt% before 10 h of hydration. With the hydration time prolonged to 3 d (Figure 14), the denser morphology appeared in these four samples. Apparently, a denser microstructure was presented in PC-CSA-2.0 wt%, which was in agreement with the data on the compressive strength and pore structure. It confirmed that the combined use of CSA and NCS contributes to the continued rapid development of the early strength of PC.

### 3.7. Discussion

The synergistic early strength enhancement of PC by CSA and NCS was found in this study, which had a more intense effect on promoting the strength development of PC than using CSA or NCS alone. Based on the above experiments, the mechanism was closely connected with the alteration of CSA and NCS on the hydration process of PC, which could be subdivided into three stages.

The first stage occurred around the range of 0–10 h, during which the CSA and NCS contribute to early strength in common. The early strength enhancement of CSA was related to the main mineral, ye’elimite (C_4_A_3_$\overset{—}{S}$), which could be rapidly hydrated to generate ettringite (0–3 h) in the presence of the calcium sulphoaluminate phase [33]. Meanwhile, the precipitation of C-S-H gel and portlandite (CH) was advanced due to the consumption of partial-free water by the fast formation of ettringite [63]. In addition, NCS also acted as extra nucleation seeds for C-S-H, diminishing the dissolution barrier of C_3_S and accelerating the large generation of C-S-H and CH. Meanwhile, the high content of CH was also beneficial to the generation of ettringite (reaction 2). Thus, the increase in the BW content of PC-CSA-2.0 wt% was higher than the sum of PC-CSA and PC-2.0 wt% at 10 h compared to PC (Table 6). It was concluded that the synergistic effect of CSA and NCS could promote the generation of hydrate, which was the main reason for the high early strength obtained by PC-CSA-2.0 wt% within 10 h. These analyses were also supported by the data of hydration heat, XRD, and TGA measurements in Figure 7, Figure 9 and Figure 10, respectively.

The second stage was between 10 and 27 h. According to the previous literatures [34] and the hydration heat analysis, the continued generation of ettringite in the PC-CSA binder would hinder the further hydration of C_3_S. Therefore, the hydration cumulative heat increased rapidly for PC and PC-2.0 wt%, while it increased slowly for PC-CSA and PC-CSA-2.0 wt% in this period. At around 27 h, the hydration cumulative heat of PC-2.0 wt% has nearly converged to the same as PC-CSA (Figure 7). For PC-CSA-2.0 wt%, a slightly higher cumulative hydration heat for PC-CSA-2.0 wt% than PC with CSA or NCS alone at 27 h was observed in Figure 6 due to the early synergistic effect of CSA and NCS. Thus, the 1 d relative compressive strength increase in PC-CSA-2.0 wt% was only slightly higher than the sum of PC-CSA and PC-2.0 wt%, which was smaller than the 10 h increase.

The third stage was between 27 and 72 h. In this period, the retardation of ettringite on the hydration of C_3_S disappeared. Thus, PC-CSA and PC-CSA-2.0 wt% exhibited a higher cumulative heat of the hydration rate than PC and PC-2.0 wt%. Meanwhile, a higher cumulative heat was observed in PC-CSA-2.0 wt% in comparison with PC-CSA. It suggested that NCS dispersed in the pore solution still could perform a seeding effect to promote the generation of inner C-S-H and CH. In turn, the high content of CH was also beneficial to promote the generation of ettringite. Thus, the presence of a "shoulder" heat flow peak at PC-CSA-2.0 wt% was observed during this period in Figure 7. This analysis was also confirmed by the TGA and XRD measurements. Thus, PC-CSA-2.0 wt% still maintained a high rate of intensity growth at 3 d.

Moreover, the micro-filling effect of NCS in the hardened PC-CSA binders was also not ignored. As shown in Figure 3 and Figure 7, the compressive strength of the PC-CSA sample at 3 d was lower than PC, but the cumulative hydration heat was higher at 3 d. Combined with the results of the MIP measurement, this contradictory phenomenon occurs because the rapid generation of ettringite increases the porosity of the PC-CSA hardened paste, leading to a breakdown in the strength development, which was in agreement with the literature [63]. However, the less porosity and denser microstructure of PC-CSA-2.0 wt% at 3 d were observed from the MIP data in Figure 12 and the SEM measurement in Figure 14. Thus, it suggested that the voids due to the fast formation of ettringite could be filled by the NCS that did not act as the nucleation sites for C-S-H gel, which solved the slow development of PC-CSA cement.

Thus, the synergistic early strength enhancement effect of CSA and NCS on PC was confirmed in this paper, and the synergistic mechanism was different at different hydration times. This study would provide a novel view of the early strength development rapidly of PC under an ambient temperature. Meanwhile, we are presently investigating the best mixing rate of NCS and CSA in PC, and additional in-depth research is expected in the future.

## 4. Conclusions

In this paper, the nano-C-S-H seed (NCS) was successfully prepared using the “co-precipitation” method. It was found that it has a better early strength enhancement effect on PC-CSA. The synergistic early strength enhancement effect of NCS and CSA on PC was confirmed. In addition, the synergy mechanism between NCS and CSA was described in detail based on the above experiment data. Specifically, the following conclusion can be drawn.

(1) CSA and NCS performed a significant synergistic effect in promoting the early compressive strength of PC paste. When CSA (replace 5% of PC) and NCS (2.0 wt%) were co-added to PC, the compressive strength of cement pastes at 10 h, 1 d, and 3 h increased by 326.3%, 52.9%, and 29.2%, respectively. In comparison with the alone addition of CSA or NCS, a higher strength enhancement was obtained by the addition of them in common.
(2) According to the hydration heat, XRD, and TGA measurements, the addition of CSA and NCS in common accelerated the hydration heat flow and cumulative heat of PC within 72 h, promoting the continuous generation of ettringite and C-S-H gel.
(3) The MIP and SEM test showed that the addition of CSA and NCS significantly reduced the porosity in the cement pastes cured at 10 h and 3 d curing times, improving the compactness of the cement matrix.
(4) The synergistic early strength enhancement of CSA and NCS was related to multiple reasons. During the initial hydration of PC-CSA-2.0 wt%, the fast precipitation of ettringite and C-S-H gel contributed to the quick formation of early compressive strength and provided a high alkali environment. With the hydration prolonged, the high concentration of CH was beneficial to the continuously generated ettringite. In addition, the NCS also performed the micro-filling effect in the PC-CSA system, which reduced the porosity of hardening cement paste and was beneficial to the strength, continuing the increment.

In conclusion, this discovery has great application prospects in precast concrete, winter construction, repair engineering, and other projects that required a high early strength of cement. It could effectively reduce steam curing, which is important for reducing the carbon emissions of the cement industry.

## Figures and Tables

**Table 1:** Chemical composition of cement (wt.%).

Code	CaO	SiO_2_	Al_2_O_3_	Fe_2_O_3_	MgO	SO_3_	TiO_2_	Na_2_O	K_2_O	LOI
PC clinker	65.74	19.64	3.68	4.18	2.28	1.27	0.18	0.32	0.88	1.33
CSA clinker	42.68	5.25	34.58	2.71	1.67	10.14	1.86	-	0.4	0.41
Anhydrite	43.53	0.5	0.1	0.2	-	55.16	-	-	-	0.32

**Table 2:** Main mineral composition of PC and CSA clinker (wt.%).

Mineral	PC Clinker	CSA Clinker
C_3_S	57.23	-
C_2_S	20.11	15.75
C_3_A	6.26	-
C_4_AF	10.83	5.23
C_4_A_3_$\overset{—}{S}$	-	68.91
C_12_A_7_	-	2.56
other	5.57	7.55

**Table 3:** Mix proportions of the cement used.

Code	PC Clinker (% Mass)	CSA Clinker (% Mass)	Anhydrite (% Mass)	NCS (wt%)
PC	95	0	5	0
PC-0.5 wt%	95	0	5	0.5
PC-1.0 wt%	95	0	5	1.0
PC-2.0 wt%	95	0	5	2.0
PC-CSA	90	5	5	0
PC-CSA-0.5 wt%	90	5	5	0.5
PC-CSA-1.0 wt%	90	5	5	1.0
PC-CSA-2.0 wt%	90	5	5	2.0

**Table 4:** The amounts of different components in the compressive strength experiment.

Code	PC (g)	CSA (g)	Anhydrite (g)	Water (g)	NCS Suspensions (g)
PC	285	0	15	90.0	0
PC-0.5 wt%	285	0	15	76.5	15
PC-1.0 wt%	285	0	15	63.0	30
PC-2.0 wt%	285	0	15	36.0	60
PC-CSA	270	15	15	90.0	0
PC-CSA-0.5 wt%	270	15	15	76.5	15
PC-CSA-1.0 wt%	270	15	15	63.0	30
PC-CSA-2.0 wt%	270	15	15	36.0	60

**Table 5:** The amounts of different components in the hydration heat experiment.

Code	PC(g)	CSA(g)	Anhydrite (g)	Water (g)	NCS Suspensions (g)
PC	2.85	0	0.15	0.90	0
PC-2.0 wt%	2.85	0	0.15	0.36	0.6
PC-CSA	2.70	0.15	0.15	0.90	0.3
PC-CSA-2.0 wt%	2.70	0.15	0.15	0.36	0.6

**Table 6:** The compressive strength growth ratio of PC with the addition of CSA or/and NCS.

Code	The Corresponding Compressive Strength Increase (%)			
	10 h	1 d	3 d	28 d
PC-0.5 wt%	28.9	9.7	−0.9	−0.3
PC-1.0 wt%	42.1	14.9	2.7	−1.5
PC-2.0 wt%	81.6	22.0	0.9	0.7
PC-CSA	157.9	14.4	−4.8	−8.8
PC-CSA-0.5 wt%	223.7	34.4	7.5	−2.4
PC-CSA-1.0 wt%	273.7	40.2	15.1	2.2
PC-CSA-2.0 wt%	326.7	52.9	29.2	0.4

**Table 7:** The generation of BW and CH (g/100 g of the dry weight of cement pastes at 550 °C).

Binder	Generation of BW			Generation of CH		
	10 h	1 d	3 d	10 h	1 d	3 d
PC	3.89	9.11	13.24	1.35	7.44	13.75
PC-2.0 wt%	5.69	9.15	13.07	3.43	7.05	13.47
PC-CSA	9.95	12.79	15.74	7.25	10.28	12.42
PC-CSA-2.0 wt%	12.36	15.22	17.62	7.44	10.55	12.23

## Data Availability

All research data can be found in this paper.
